# Thermotolerant yeasts promoting climate-resilient bioproduction

**DOI:** 10.1093/femsyr/foaf047

**Published:** 2025-09-10

**Authors:** Roghayeh Shirvani, Maryam Babaei, Motahare Baladi, Matthias G Steiger, Mohammad Barshan-tashnizi

**Affiliations:** Institute of Chemical, Environmental and Bioscience Engineering, TU Wien, 1060 Vienna, Austria; Department of Bioengineering, School of Life Science Engineering, College of Interdisciplinary Science and Technology, University of Tehran, 1439957131 Tehran, Iran; Department of Bioengineering, School of Life Science Engineering, College of Interdisciplinary Science and Technology, University of Tehran, 1439957131 Tehran, Iran; Institute of Chemical, Environmental and Bioscience Engineering , TU Wien, 1060 Vienna, Austria; Department of Bioengineering, School of Life Science Engineering, College of Interdisciplinary Science and Technology, University of Tehran, 1439957131 Tehran, Iran

**Keywords:** sustainable bioproduction, global warming, *Komagataella phaffii*, *Saccharomyces cerevisiae*, methylotrophic yeasts, heat-shock response

## Abstract

The growing challenges posed by global warming and the demand for sustainable food and feed resources underscore the need for robust microbial platforms in bioprocessing. Thermotolerant yeasts have emerged as promising candidates due to their ability to thrive at elevated temperatures and other industrially relevant stresses. This review examines the industrial potential of thermotolerant yeasts in the context of climate change, emphasizing how their resilience can lead to more energy-efficient and cost-effective bioprocesses. Particular attention is paid to the thermodynamic implications of yeast metabolism under heat stress, especially in bioethanol production and methanol metabolism in methylotrophic yeasts, where metabolic heat generation plays a critical role. The cellular and molecular mechanisms underlying thermotolerance are also reviewed, including heat shock sensing mechanisms, the protection of biomolecules, and membrane and cell wall integrity. Advances in genetic and metabolic engineering aimed at enhancing these traits are also highlighted. By integrating current insights into the molecular and cellular mechanisms of thermotolerance, along with recent technological advancements, this review outlines the advantages of high-temperature operations and positions thermotolerant yeasts as vital components of future sustainable bioproduction systems.

## Teaser

This review positions thermotolerant yeasts as promising, climate-resilient biocatalysts for sustainable bioproduction, highlighting their energy-efficient industrial applications, molecular thermotolerance mechanisms, and engineering advances to overcome thermodynamic challenges in alcohol metabolism.

## Introduction

Since the Industrial Revolution, the global surface temperature has risen by roughly 1.1°C. This trend is predicted to continue without a major decrease in greenhouse gas emissions. According to the most recent IPCC Assessment Report (AR6)(www.ipcc.ch), there is a higher than 50% risk that the global temperature rise would exceed 1.5°C (2.7°F) between 2021 and 2040. On the other hand, the human population on Earth is expected to reach 9–11 billion by 2050 (Tomiyama et al. 2020). The world population growth, combined with climate change, is having significant impacts on food resources and food security, driving up overall food demand. It is predicted that warming trends will likely reduce global agricultural yields by ~1.5% per decade without effective adaptation measures (Frank et al. 2017, Malhi et al. 2021). Therefore, global warming and related climate changes can result in substantial economic losses and an increase in the number of undernourished people (Wheeler and Von Braun 2013).

Addressing the challenges posed by climate change to agriculture and food security requires innovative solutions, such as the utilization of beneficial microbes to enhance production and environmental sustainability. Industrial microorganisms, including bacteria, fungi, and algae, play a central role in producing biomass and bioproducts. They can grow on diverse substrates, ranging from agricultural residues to industrial byproducts and syngas products resulting from gasification (Frazão and Walther 2020, García Martínez et al. 2022, Shirvani et al. 2023).

Thermotolerant microbes have undergone evolutionary adaptations to withstand high-temperature habitats in nature. As a result of their unique biochemical properties, these thermotolerant microbes emerge as highly promising candidates for various industrial applications (Gavande et al. 2021, Yamini et al. 2022, Okoro et al. 2023).

Altogether, global warming could be viewed from two angles from biotechnology prospects: first, its impact on natural environments, particularly microbial populations within the ecosystem. Second, industrial bioproduction, notably as the demand for sustainable food sources grows in the face of global warming. In a recently published work, Sitara et al. (2024) reviewed the vital role of thermoanaerobic bacteria in promoting sustainable bioprocesses and advancing the circular carbon economy, contributing to efforts to combat climate change.

This review highlights the importance of thermotolerant yeasts in current and future industrial bioproduction. Furthermore, we have outlined how lessons from microbial adaptation can guide the engineering of thermotolerant yeast strains.

## Thermophilic or thermotolerant, which term fits the heat resistance in yeasts?

The definition of thermotolerance in microorganisms varies depending on the topic and condition under study. A few yeasts were also reported to be obligatory psychrophilic, found in Arctic environments, with a maximum temperature for growth of 20°C (Watson et al. 1978), such as *Rhodotorula frigidialcoholis* and *Cryptococcus* spp. (Liu et al. 2023). Most of the yeast species employed in biotechnology are mesophilic, meaning their optimal growth temperature (T_opt_) is around 30°C (Segal-Kischinevzky et al. 2022), such as brewer’s yeast, *Saccharomyces cerevisiae* (Lip et al. 2020), methylotrophic yeasts *Komagataella phaffii* and *Candida boidinii* (Volfová et al. 1993), and oleaginous yeasts such as *Yarrowia lipolytica* (Patsios et al. 2020) and *Rhodotorula glutinis* (Saenge et al. 2011, Mussagy et al. 2021). Only a few yeast species can grow at temperatures above 45°C, such as *Ogataea polymorpha* (Vasylyshyn et al. 2024) and *Kluyveromyces marxianus* (Karim et al. 2020), which is still in the range of the lower limit of the growth temperature of thermophilic prokaryotes. Eukaryotes face an upper temperature threshold near 60°C due to structural constraints stemming from their inability to maintain functional and structurally stable organellar membranes at elevated temperatures (Tansey and Brock 1972).

Publications on yeast physiology use slightly different definitions of thermophilic and thermotolerant in yeast, although they are all based on minimum (T_min_) and maximum (T_max_) growth temperatures determined by experimental methods and mathematical modeling. T_min_ is the lowest limit of the biokinetic temperature range for a given microbe or microbial community, whereas T_max_ is the maximum temperature at which a microorganism could grow (Moro and Muga 2006, Huang et al. 2011). Watson et al. (1978) described thermophilic yeasts with a T_min_ above 20°C and no restriction in T_max_. Later, Mouchacca (1997) defined thermophilic fungi as those with a T_max_ of 50°C or higher and a T_min_ of 20°C or higher. Satyanarayana and Kunze (2009) also considered thermotolerant yeasts as those that can grow at temperatures above 20°C.

In summary, thermophilic yeasts have a minimum growth temperature of 20°C and thrive at high temperatures, with a range from 20°C to ≥46°C, rarely up to 50°C. In contrast, thermotolerant yeasts grow across a broader range, including below 20°C, and tolerate temperatures up to 50°C. Although thermotolerant yeasts can survive and function at elevated temperatures, their growth rates and metabolic performance at extreme heat are generally lower than those of thermophilic yeasts, which are better adapted to such conditions.

Given the above definitions, thermophilic yeasts that can grow above 50°C are relatively rare and poorly known, such as *Ogataea thermophila* and *Takashimella tepidaria* (Segal-Kischinevzky et al. 2022). A few yeasts reported to thrive around 48°C–50°C have a T_min_ below 20°C, and therefore, they are considered thermotolerant (Buzzini et al. 2017, 2018).

## Thermotolerance and pathogenicity

The gradual rise in global temperatures due to climate change significantly impacts microbial communities, resulting in changes to biodiversity and the redistribution of pathogens (Hutchins et al. 2019, Ibáñez et al. 2023). While thermotolerant microbes offer significant advantages for various applications, their use might pose potential risks that must be carefully considered. Genetic modifications to microorganisms to enhance thermotolerance might also increase pathogenic characteristics (Karavolias et al. 2021, Rokas 2022). To mitigate these risks, safety protocols, including risk assessments, continuous monitoring, and documentation of microbial strains through adherence, are crucial (Kimman et al. 2008). A safety-by-design approach could also be applied, e.g. using genetic safeguards such as auxotrophy (Directorate-General for Health and Consumers—European Commission 2015, Asin-Garcia et al. 2023).

Addressing this topic, Fisher et al. (2022) suggested that global warming may contribute to the emergence of resistance to the antifungal agents in fungi by driving environmental changes, such as increased temperatures. Such conditions favor the selection of fungi with enhanced thermotolerance, enabling them to adapt more readily to mammalian body temperatures (~37°C). This adaptation may, in turn, increase their pathogenic potential. Furthermore, rising temperatures could extend the geographical range and infectivity of pathogenic fungi such as *Aspergillus fumigatus* and *Candida* species, thereby increasing the risk of fungal infections in humans and animals (Fisher et al. 2022).

*Candida* is a diverse, polyphyletic genus of yeasts, encompassing over 400 species across nearly all families of the Saccharomycotina subphylum, with about 42% of 810 known strains being thermotolerant and able to grow at 37°C or above (Robert et al. 2015, Liu et al. 2024). An example of a well-known thermotolerant pathogenic yeast fungus is *Candida albicans*, which can grow at human body temperature (35°C–40°C) (Leach et al. 2012, Mayer et al. 2013). The heat shock response (HSR) and thermal adaptation of *C. albicans* have been extensively studied, as evidenced by research on its heat shock proteins (HSPs) and thermal regulation mechanisms (Guo et al. 2009). However, other than its thermal tolerance, *C. albicans* possesses multiple virulence factors that contribute to its pathogenicity, such as adhesion to the host cell, biofilm formation, and hydrolytic enzymes (Talapko et al. 2021).

The multidrug-resistant yeast *Candida auris* serves as an example of how climate change can affect the emergence of new pathogen microbes (Casadevall et al. 2019, Misseri et al. 2019, De Gaetano et al. 2024). *Candida auris* evolved from an environmental fungus to a human pathogen, aided by increased thermal and salinity tolerance and overexpression of HSP HSP90 (Casadevall et al. 2019). It was speculated that its transformation from a plant saprophyte to a human pathogen was aided by global warming as rising temperatures and UV radiation enhanced its survival in human hosts (Ellwanger and Chies 2022, Garcia-Bustos 2024).

Although the ability to grow at 37°C is required for human pathogenic fungi, this behavior is not exclusive to pathogenic strains. Some nonpathogenic fungi can also thrive at high temperatures, even those that have generally recognized as safe (GRAS) status applications in the industrial sector. This topic is discussed in the following section.

## Thermotolerant yeasts in bioprocesses, unlocking the advantages of operation at high temperature

The rise in global temperatures has necessitated the investigation of novel ways within industrial processes to improve efficiency and promote sustainability. Thermotolerant yeasts could be a viable option in these circumstances. However, there are only a few naturally thermotolerant yeast species used in biotechnology.

*Ogataea polymorpha* (formerly known as *Hansenula polymorpha*) is a methylotrophic yeast with a relatively high optimal growth temperature (37°C–43°C) (Satyanarayana and Kunze 2009) and a heat tolerance of up to 50°C (Vasylyshyn et al. 2024). High-density fermentation was reported for the *O. polymorpha* strains, while it can assimilate a broad substrate utilization spectrum, including glucose, xylose, and methanol (Ryabova et al. 2003, Tsuda and Nonaka 2024, Xie et al. 2024). *Ogataea polymorpha* is mostly used to produce recombinant proteins, particularly in the pharmaceutical industry for manufacturing insulin, hepatitis B vaccinations, interferon alpha (Gellissen 2002, Xie et al. 2024), and mammalian proteins (Manfrão-Netto et al. 2019).

*Kluyveromyces marxianus* is another thermotolerant yeast that thrives at temperatures exceeding 40°C. *Kluyveromyces marxianus* was mostly isolated from food and beverage environments (Varela et al. 2017, Karim et al. 2020). This yeast is reported to be used not only in high-temperature procedures such as bioethanol production but also as a host for heterologous protein and single-cell protein (SCP) production (Fonseca et al. 2008). Its ability to metabolize lactose and produce ethanol at higher temperatures makes it a suitable industrial yeast for converting dairy waste and other biomass into biofuels (Lehnen et al. 2019, Montini et al. 2022).

*Blastobotrys (Arxula) adeninivorans* is a highly robust, nonconventional yeast increasingly recognized in industrial biotechnology for its thermotolerance, with optimal growth at 30°C–40°C, tolerance up to 48°C, and survival at 55°C for some hours (Kunze and Kunze 1996, Malak et al. 2016, Bischoff et al. 2017). *Blastobotrys adeninivorans* is noted for its morphological plasticity, which allows it to switch between yeast and hyphae forms depending on temperature, with yeast forms at lower temperatures and (pseudo)hyphal forms above 42°C (Thomas et al. 2019). This yeast exhibits a versatile substrate range, metabolizing glucose, xylose, adenine, and purine derivatives, making it suitable for bioprocessing agricultural and industrial byproducts (Malak et al. 2016, Thomas et al. 2019). *Blastobotrys adeninivorans* is widely used for producing recombinant proteins, such as enzymes and biopharmaceuticals, due to its efficient secretion system and genetic tractability (Kunze et al. 2014, Malak et al. 2016).

*Schizosaccharomyces pombe* (known as fission yeast) is also used in the industrial-scale fermentation of sugarcane molasses into bioethanol and alcoholic fermentation of wines due to its fermentation efficiency, ease of genetic manipulation, and ability to thrive in diverse environments (Loira et al. 2018, Vassiliadis et al. 2020). It has been reported that *S. pombe*, although not naturally recognized as thermotolerant, exhibits a degree of thermal resistance through adaptive responses to mild heat shocks. Its stationary phase cultures can survive high temperatures, even at 55°C, controlled by a circadian rhythm (Kippert 1989, Fernández et al. 1997).

The conventional yeast *S. cerevisiae* is not inherently considered a thermotolerant yeast, as its optimal growth temperature is typically at 28°C–33°C (Lip et al. 2020). However, it can develop thermotolerance features through adaptive evolution and genetic engineering. Industrial strains of *S. cerevisiae* have been engineered or evolved to withstand temperatures above 40°C, which is beneficial for processes like bioethanol production (Shui et al. 2015, Yang et al. 2023, Zhang et al. 2023). *Saccharomyces boulardii*, which is often considered a variant of *S. cerevisiae*, is a thermotolerant yeast that grows optimally at 37°C (Czerucka et al. 2007, Hossain et al. 2020, Singu et al. 2020). Indeed, *S. boulardii* is the only GRAS-commercialized yeast used as a probiotic for treating diarrhea and gastrointestinal disorders (Hossain et al. 2020).

The ability of thermotolerant yeast cells to grow well at higher temperatures does not necessarily guarantee optimal production yield of desired products under the same conditions. Elevated temperatures can impair key metabolic enzymes, causing a slowdown in overall yeast metabolism. Stress responses divert resources toward survival, such as synthesizing HSPs and stabilizing cellular structures. Consequently, production yield is sacrificed for cell viability. Studies show that yeast strains differ in their ability to handle combined thermal and ethanol stresses, leading to different growth patterns and protein production levels. For instance, in *S. cerevisiae*, ethanol yields may decrease at high temperatures (e.g. >40°C) due to increased ethanol toxicity and metabolic stress, which impair fermentation efficiency. High temperatures trigger stress responses, upregulating genes such as *SOD1* (antioxidant defense), *PDA1, CIT1, PDC1, ADH1* (ethanol metabolism), and HSPs (e.g. HSP70 and HSP104) to maintain cell viability. These responses may divert metabolic resources or slow down central metabolism, potentially impacting ethanol production (Fonseca et al. 2008, Kim et al. 2013, Phong et al. 2022).

On the other hand, Khatun et al. (2017) reported that the thermotolerant *S. cerevisiae* strain MNII-AZFP achieved significantly higher ethanol yields at 42°C, producing 8.7 g/l from phosphoric acid-swollen cellulose (PASC) and 28.2 g/l from Jerusalem artichoke stalk (JAS), compared to 1.9 g/l and 1.0 g/l, respectively, for a control strain. Also, fermentation efficiency improved, with a 19% higher ethanol yield from PASC and a 15% higher yield from JAS compared to the control, alongside a 12-hour reduction in fermentation time for JAS (Khatun et al. 2017).

In recombinant protein-producing yeasts, production-related stresses resemble thermal stress, and higher temperatures often reduce protein yields due to impaired folding and secretion. While enhancing thermotolerance can improve production, the relationship is complex, and yeast must simultaneously optimize energy supply and stress resistance. Genetic modifications such as the CYR1 mutation in *K. marxianus* improve both thermotolerance and protein yield by regulating cAMP signaling cascades at high temperatures, such as 46°C (Ren et al. 2024).

Some engineered yeast strains can perform well at high temperature (>40°C) for short fermentations, but may subsequently lose essential genetic material or show variability in recombinant expression if continued longer. This instability has major implications for industrial reliability, batch-to-batch consistency, and regulatory compliance. Studies highlight that optimal growth and genetic stability in yeast are achieved at 25°C–35°C. Deviating above this range accelerates mutation rates and genetic instability substantially, requiring careful monitoring and possibly reengineering for robust industrial use at higher temperatures (Huang et al. 2018, Li et al. 2024).

Heat shock and temperatures above the normal range for yeast (25°C–35 °C) can dramatically enhance rates of chromosomal aberrations, mitotic recombination, and even aneuploidy (abnormal chromosome numbers). Controlled experiments show that brief exposures (minutes) to extreme temperature (e.g. 52°C) increase recombination by an order of magnitude and chromosomal abnormalities (Shen et al. 2020). Elevated temperatures can also challenge the stability of genomically integrated cassettes (not just episomal/plasmid DNA), leading to loss or reduced expression of the recombinant gene. This instability may arise from stress-induced DNA repair mechanisms or increased rates of mitotic recombination and transposon mobilization, which may excise or silence integrated sequences. Shalguev et al. (2004) showed that the Rad51 protein from the thermotolerant yeast *O. polymorpha* exhibits higher thermostability compared to that from nonthermotolerant yeasts, maintaining recombination activity at temperatures up to 54°C. This protein’s thermostability likely contributes to genetic stability during DNA repair and recombination processes at elevated temperatures, supporting the overall genetic stability of thermotolerant yeasts under heat stress (Shalguev et al. 2004).

Industrial applications seek strains that combine robust thermotolerance with high production efficiency, which remains challenging due to the trade-offs between stress survival and metabolic productivity. While thermal growth tolerance enables yeast survival at elevated temperatures, maintaining high production performance (ethanol or recombinant proteins) under these conditions requires additional adaptations and modifications to optimize metabolic flux, energy status, stress response pathways, and maintain genetic stability. The two traits, though related, are not directly proportional, and engineering or selecting strains with both qualities is a major focus in industrial microbiology (Kim et al. 2013, Khamwachirapithak et al. 2023, Ren et al. 2024).

## Thermodynamics of yeast metabolism

Understanding yeast thermodynamics enables better control of energy consumption and heat management in industrial fermentation. Considering power and energy requirements is not only essential for scaling up but also for assessing the environmental footprint and sustainability of bioprocesses (Cardoso et al. 2020). The total energy consumption over time is determined by integrating the power usage (Knoll et al. 2007). The power input in stirred-tank bioreactor cultivation is primarily defined as the energy transferred to the liquid medium per unit of time, mainly through mechanical agitation and aeration. Additional power is also required for temperature control, pumping, and other auxiliary operations (Kaiser et al. 2018, Müller et al. 2018, Schirmer et al. 2021). The cooling capacity depends on both the metabolic heat generated by the growth of the microorganisms and the heat transferred from the environment. This means that the operating temperature also depends on the geographical region, which leads to a significant energy cost (Kumar et al. 2013, Petříček et al. 2018).

Many industrial applications, such as the production of enzymes, proteins, and other metabolites, often use aerobic conditions. When considering the heat generated during yeast growth under aerobic conditions, two main factors must be taken into account: the heat released from substrate oxidation and the heat associated with biomass formation. The heat released during biological oxidation processes, such as cellular respiration (Equation 1), can be estimated using the standard molar heat of combustion (Δ*h*_c_°) of biomolecules (Equation 2). Δ*h*_c_° mainly depends on the elemental composition of the biomass and the carbon source, particularly its carbon, hydrogen, and oxygen content. However, the elemental composition of yeast biomass remains generally consistent across species and substrates. For instance, the methylotrophic yeast *C. boidinii* grown on glucose has a heat of combustion of 20.14 ± 0.18 kJ/g, while it is 21.51 ± 0.09 kJ/g when grown on methanol (Cordier et al. 1987, Doran 2013). Meanwhile, the heat of combustion of *S. cerevisiae* biomass, grown in batch culture on glucose, has been measured by calorimetry as 21.39 ± 0.33 kJ/g (Larsson et al. 1993). Using the Δ*h*_c_° values of the combustible reactants and products, the reaction enthalpy (ΔH_rxn_) can be calculated based on Hess’s Law (Equation 3). This represents the heat released during substrate oxidation under ambient conditions (Doran 2013). Alternatively, for fully aerobic reactions, where molecular oxygen acts as the electron acceptor, a simplified estimation method can be used (Equation 4) (Cooney et al. 1969). The exothermic nature of biological reactions for cell growth (ΔH_rxn _< 0), such as ethanol fermentation and methanol oxidation, is another reason that fermentation vessels often require cooling to maintain optimal temperatures for yeast growth.


(1) \begin{eqnarray*}
&& {{C}_w}{{H}_x}{{O}_y}{{N}_z} + a {{O}_2} + b {{H}_g}{{O}_h}{{N}_i}\\
&&\quad \, \to c C{{H}_\alpha }{{O}_{\beta}}{{N}_i} + d C{{O}_2} + e {{H}_2}O.\quad
\end{eqnarray*}

(Doran 2013).


(2) \begin{eqnarray*}
\Delta {{h}_c}^\circ \ = \ - q\gamma {{x}_C}.
\end{eqnarray*}

(Doran 2013). *q = *The heat evolved per mole of available electrons transferred to oxygen *γ =* The degree of reduction of the compound *x_C_ = *The number of carbon atoms in the molecular formula


(3) \begin{eqnarray*}
{\mathrm{\Delta H_{rxn} \ }} = {\mathrm{\ }}\mathop \sum \limits_{\textit{substrates}} n{\mathrm{\Delta }}h{\mathrm{c}}^\circ - \mathop \sum \limits_{\textit{products}} n{\mathrm{\Delta }}h{\mathrm{c}}^\circ.
\end{eqnarray*}

(Doran 2013).


(4) \begin{eqnarray*}
&& {\mathrm{\Delta H_{rxn} \ for\ aerobic\ metabolism\ }} \\
&&\quad = {\mathrm{\ }} - 460{\mathrm{\ kJ\ mo}}{{{\mathrm{l}}}^{ - 1}}{\mathrm{\ per\ mole\ of\ O}}_{ 2}{\mathrm{\ consumed}}.
\end{eqnarray*}

(Cooney et al. 1969).


(5*) \begin{eqnarray*}
{{C}_6}{{H}_{12}}{{O}_6} + 6 {{O}_2} \to 6 C{{O}_2} + 6 {{H}_2}O\,\Delta {{H}_{rxn \approx }} - 2760\,kJ/mol\ of \ \textit{glucose}.\\
\end{eqnarray*}

(6†) \begin{eqnarray*}
{{C}_6}{{H}_{12}}{{O}_6} \to 2 {{C}_2}{{H}_5}OH + 2 C{{O}_2}\Delta {{H}_{rxn = }} - 70\ kJ/mol\ of \ \textit{glucose}.\\
\end{eqnarray*}

(7*) \begin{eqnarray*}
C{{H}_3}OH + 3/2 {{O}_2} \to C{{O}_2} + 2 {{H}_2}O\Delta {{H}_{rxn \approx }} - 690\ kJ/mol \ of \ \textit{methanol}.\\
\end{eqnarray*}

(8*) \begin{eqnarray*}
C{{H}_3}OH + {{O}_2} \to C{{H}_2}O + {{H}_2}{{O}_2}\Delta {{H}_{rxn \approx }} - 460\ kJ/mol \ of \ \textit{methanol}.\\
\end{eqnarray*}

*The approximate ΔH_rxn_ of aerobic reaction using oxygen as an electron acceptor was determined based on Equation (4).

†ΔH_rxn_ of anaerobic glucose fermentation into ethanol was determined using standard heats of combustion values of the combustible reactants (Haynes et al. 2014) and products based on Equation (3).

### Heat generation in ethanol fermentation

The most prominent bioethanol production processes encompass the preparation of the feedstock, its fermentation, distillation, and dehydration. Second-generation bioethanol production utilizes lignocellulosic biomass, requiring processes such as feedstock preparation, fermentation, and subsequent ethanol recovery. Efficient fermentation of sugars derived from cellulose and hemicellulose, such as glucose and xylose, typically necessitates genetically engineered yeast strains, such as *S. cerevisiae*, optimized for enhanced sugar metabolism. These components undergo pretreatment and enzymatic hydrolysis to release fermentable sugars, which are then converted into ethanol during fermentation (Cámara et al. 2022, Asemoloye et al. 2023, Vasylyshyn et al. 2025).

The approximate standard enthalpy of combustion for 1 mol of glucose completely oxidized to carbon dioxide and water is −2760 kJ/mol (Equation 5). However, due to the Crabtree effect, complete oxidation of glucose to CO_2_ by respiration does not often occur in *S. cerevisiae* cultures when glucose is abundant. Instead, *S. cerevisiae* ferments glucose to ethanol and CO_2_ (González-Hernández et al. 2024). Although ethanol fermentation produces less heat than complete glucose oxidation (70 kJ per mole of glucose), it is still an exothermic process (Equation 6). Therefore, as *S. cerevisiae* is a mesophilic yeast, an effective cooling system are essential to regulate fermentation temperatures, minimize yeast stress, and maintain product consistency (Deparis et al. 2017, Schwinn et al. 2019).

An online calorimetric set-up was integrated into the bioreactor system for real-time measurement of heat produced by *S. cerevisiae* culture. A significant heat flow rate of 65 W/l was observed at the end of the 32 h fed-batch cultivation in a 30 l stirred-tank bioreactor in a fed-batch fermentation process (Biener et al. 2012). Typically, fermentation tank cooling jackets are integrated into the outer walls of fermentation tanks, circulating coolant (usually cold water or ethylene glycol solution) to absorb heat generated by yeast metabolism. A combination of jacketed vessel recirculating chillers may be required to maintain the optimal temperature range (Junker et al. 2003, Coetzee et al. 2024). The cooling water is thought to enter the bioreactor heat transfer system at 20°C and exit at 25°C. To cool down to 20°C, a vapor compression refrigeration cycle was employed (Fitzpatrick 2019). However, in tropical regions, the fermentation temperature was kept at 32°C by using cooling water at 28°C, leading to ~40% of the total energy consumption in fermentation and distillation processes being attributed to removing heat from the bioreactor (Kumar et al. 2013). According to another case study on the manufacture of ethanol with *S. cerevisiae*, ~550 m^3^ of cooling water at 25°C is needed for every m^3^ of ethanol in order to keep the temperature at 32°C for a 32 h fermentation period. About 70 kW/h is needed to run the cooling towers and circulate water to the bioreactor. Overall, it was found that the energy required for the fermentation and distillation is 12.63 MJ/l of ethanol (Khatiwada and Silveira 2009).

The use of thermotolerant yeast in bioethanol production can significantly reduce cooling costs and the risk of contamination while enabling simultaneous saccharification and fermentation, offering clear advantages over mesophilic strains (Ballesteros et al. 1991, Kosaka et al. 2019). Thermotolerant yeasts, such as *Pichia kudriavzevii* and heat-resistant strains of *S. cerevisiae*, are recommended for effectively fermenting ethanol at temperatures as high as 45°C (Talukder et al. 2016). *Kluyveromyces marxianus* was also used for bioethanol production at high temperature (Yanase et al. 2010, Arora et al. 2015). It was found that the possibility of a 5°C increase in the fermentation temperature can significantly affect the production costs, while no cooling was needed at 42°C (Abdel-Banat et al. 2010).

Thermotolerant, methylotrophic yeast *O. polymorpha* has also attracted considerable interest for bioethanol production due to its robust fermentative capabilities. It has demonstrated the ability to efficiently metabolize a variety of carbon sources, including glucose, xylose, cellobiose, and glycerol, under elevated temperature conditions, typically around 45 °C (Sibirny 2022, Vasylyshyn et al. 2024). Overexpression of the genes *PDC1* and *ADH1* in *O. polymorpha* resulted in the production of 5.0 g/l ethanol from glycerol at 45°C (Kata et al. 2016). Further overexpression of glycerol catabolism and transporter genes (such as *GUT1, GPD1, GCY1*, and *DAK1*) in *O. polymorpha* enhanced ethanol production from crude glycerol, a biodiesel by-product, reducing reliance on petroleum-based fuels. Recombinant strains achieved ethanol yields of up to 10.7 g/l from 15% pure glycerol at 37°C (15-fold higher than wild-type) and 3.55 g/l from crude glycerol, with productivities of 30 mg/g biomass/h and 1.6 mg/g biomass/h, respectively (Semkiv et al. 2019).

While most efforts to improve the thermotolerance of *S. cerevisiae* have focused on genetic and molecular approaches, cell immobilization-based strategies have also shown promise. One such method is the encapsulation of yeast cells in alginate–chitosan material (Ylitervo et al. 2011, Westman et al. 2012, Gulli et al. 2020). Ylitervo et al. (2011) showed that *S. cerevisiae* encapsulated in semipermeable alginate–chitosan capsules could perform robust fermentation at 42°C using 30 g/l glucose, with ethanol yields exceeding 80%. However, cell growth declined after several fermentation cycles (Ylitervo et al. 2011). Proteomic analysis revealed that encapsulation of *S. cerevisiae* led to the upregulation of stress response proteins such as Glc7, Hsp12, and Gre3, as well as proteins involved in the trehalose biosynthesis pathway (Westman et al. 2012).

### Heat generation in methanol metabolism

Methylotrophic yeasts can use single-carbon compounds, such as methanol, as their sole source of carbon and energy for growth. This makes them an attractive platform for developing sustainable biotechnology processes that can reduce dependence on fossil resources (Baumschabl et al. 2024, Severinsen et al. 2024). The most commonly known methylotrophic yeasts with biotechnological applications are *K. phaffii, O. polymorpha, C. boidinii*, and *Ogataea methanolica*. Among them, *O. polymorpha* stands out as a thermotolerant species that can withstand temperatures of up to 50°C (Wefelmeier et al. 2023).

During the 1970s, Phillips Petroleum Company developed media and protocols for growing *K. phaffii* (formerly known as *Pichia pastoris*) in continuous culture at high cell density, enabling its use as a host system for biomass (SCP) and recombinant protein production (Cereghino and Cregg 2000, Ergün et al. 2022). This nononventional yeast is used in bioproduction due to its efficient protein secretion, availability of constitutive and inducible promoters, and compatibility with genetic engineering tools (Ata et al. 2021). Recent developments, including the GoldenPiCS modular cloning system and CRISPR-based genome editing, have increased its potential for metabolic engineering and strain optimization (Prielhofer et al. 2017, Peña et al. 2018, Gassler et al. 2019). The optimum growth temperature of this facultative methylotrophic yeast is 28°C–32°C. The methylotrophic lifestyle is exothermic; therefore, it can bring extensive cooling energy usage while growing on methanol. The specific heat generated from the biomass formed on methanol with *K. phaffii* was calculated to be 1184 kJ/C-mol. This is 3.5 times higher than the reported heat generation in aerobic growth of *S. cerevisiae* on glucose (339 kJ/C-mol of biomass) (Curvers et al. 2001).

Methanol oxidation to formaldehyde via alcohol oxidase (AOX) (Waterham et al. 1997) is the first step in methanol utilization in *K. phaffii*. This reaction also produces hydrogen peroxide (H_2_O_2_) as a byproduct. Formaldehyde, a central intermediate, can follow two paths: first, the assimilatory pathway, which involves the xylulose monophosphate cycle, where formaldehyde is assimilated into cellular biomass. In a dissimilatory pathway occurring in the cytosol, formaldehyde is oxidized to carbon dioxide (CO_2_) in a few reaction steps, releasing energy in the form of heat (Rußmayer et al. 2015). Methanol metabolism is known to be highly exothermic (Equation 7). In methylotrophic yeast, methanol oxidation or dissimilation occurs in the cytosol and is a major source of NADH and energy production. However, when methanol is assimilated into biomass, this substrate is not fully oxidized to CO_2_. Instead, it is partitioned between formaldehyde dissimilation and assimilation. It is worth mentioning that the initial step of methanol oxidation to formaldehyde, catalysed by AOX, releases nearly two-thirds of the molecule’s energy (Equation 8).

In comparison to the methylotrophic yeasts, in most methylotrophic thermophilic bacteria, NAD^+^-dependent alcohol dehydrogenase is responsible for the first step of methanol utilization (Singh et al. 2022, Sarwar and Lee 2023). This reaction oxidizes methanol to formaldehyde and harvests electrons to NADH in only one reaction. Alcohol dehydrogenase (Adh2) is also found in *K. phaffii* as a promiscuous enzyme (Zavec et al. 2021). However, the Gibbs free energy of methanol oxidation by this reaction is not favorable at ambient temperature (Whitaker et al. 2015). This is suggested to be one of the reasons behind mesophilic methylotrophic yeasts, such as *K. phaffii* evolving peroxisomal AOX that transfers electrons to oxygen at ambient temperatures by releasing energy to the surroundings (Zavec et al. 2021). Considering the above arguments, while methylotrophic growth offers advantages for sustainable renewable energy consumption, considerable heat generation may pose challenges for industrial-scale applications. To harness the full potential of methylotrophic yeast *K. phaffii* for minimizing the environmental impact and improving overall process efficiency, several factors should be considered. In addition to process optimization for cost efficiency and the development of more effective energy management systems, strain engineering strategies to improve thermotolerance features could also be explored.

### Sustainability assessment of thermotolerant bioproduction

Designing energy-efficient processes is a principle of green chemistry and an essential part of broader sustainability strategies (Ribeiro et al. 2015, Pleissner and Kümmerer 2018). To accurately assess the benefits of thermotolerant yeasts, life cycle assessments (LCAs) and techno-economic analyses (TEAs) must be conducted, including evaluations of energy consumption (Sae-ngae et al. 2020). A TEA study on thermotolerant oleaginous yeast for microbial lipid production revealed an 85% decrease in the demand for cooling water and an 11% reduction in the cost of installing equipment by omitting chillers (Karamerou et al. 2021).

Operating at higher temperatures can reduce reliance on energy-intensive cooling systems, which are often necessary to maintain mesophilic fermentation conditions. Life cycle climate performance analyses showed chillers used in industrial scale are highly energy-intensive and typically rely on fossil fuel-based electricity, making them significant contributors to the carbon footprint of industrial processes (Akinseloyin et al. 2023).

## Molecular mechanisms of thermotolerance in yeasts

A clear understanding of the cellular and molecular mechanisms behind yeast thermotolerance could lead to the development of robust strains for industrial applications. The use of various biological and engineering approaches to construct and harness the capabilities of thermotolerant microorganisms in the production of sustainable products and services has a long history.

Although some strain-improvement methods, such as natural selection and adaptive laboratory evolution (ALE), are considered older, they are still widely utilized. These methods are random in nature and not specifically designed to target particular mechanisms. Nevertheless, they have effectively advanced the development of microbial tolerance due to their minimal technical requirements. For instance, a *K. marxianus* strain isolated from sugar mills was capable of producing 6% ethanol from 15% glucose at 47°C. This capability is the result of natural selection in tropical areas under high temperatures (Anderson et al. 1986). Caspeta and Nielsen (2015) used ALE to develop seven *S. cerevisiae* strains capable of growing at 40°C. Interestingly, these evolved strains exhibited reduced growth rates at lower temperatures (30°C–34°C), suggesting a trade-off in thermal adaptation. The maximum survivable temperature for these strains was estimated to be around 49°C, above which protein denaturation occurred significantly. The evolved strains also showed improved resistance to additional stressors, such as ethanol and reactive oxygen species (ROS), and to lower pH (Caspeta and Nielsen 2015). Salas-Navarrete et al. (2023) extended the thermo-acid tolerance profiles in *S. cerevisiae* using ALE, creating strains with shifted pH/temperature growth optima. The evolved yeast strains showed increased survival at temperatures around 40°C, while the growth of the wild-type strain was significantly reduced. They also showed higher tolerance to acetate, growing at concentrations up to 60 mM, compared to the wild-type, which showed inhibited growth at lower acetate levels (Salas-Navarrete et al. 2023). In another study, an ALE strategy with fluconazole and FK506 was used to improve the heat resistance of an industrial baker’s yeast, yielding six isolates that grew at 41.5°C (µmax: 0.07–0.2/h) (Sánchez-Adriá et al. 2025).

On the other hand, the field of synthetic biology has enabled the design and engineering of customized thermotolerant microorganisms with enhanced environmental capabilities. This is possible by strategically incorporating heat-resistant mechanisms into microbial strains. This methodology expands the variety of substrates available while enhancing the overall strength and effectiveness of bioproduction systems (Chung et al. 2010, Westfall et al. 2012, Patra et al. 2021, Logan et al. 2024).

However, engineering thermotolerance into mesophilic yeasts through the expression of a single gene is challenging due to the complex nature of the trait. A relevant study by Montini et al. (2022) revealed that KLMX_70384, a gene present in the yeast *K. marxianus*, is involved in competitive growth at high temperatures (45°C–47°C). Although this gene is known to encode a protein and exhibits some features of RNA-binding activity, its precise function remains unclear. Heterologous expression of KLMX_70384 in the mesophilic yeast *K. lactis* did not confer thermotolerance. This suggests that its functionality may be species-specific and dependent on protein interactions with a shared evolutionary history (Montini et al. 2022).

Therefore, improving yeast thermotolerance through genetic engineering requires a more accurate understanding of yeast thermotolerance mechanisms. The exposure of yeast cells to environmental changes results in the expression of both general and specific stress responses (Causton et al. 2001, Auesukaree 2017). It was shown that heat stress induces metabolic reprogramming in *S. cerevisiae*, altering its metabolic pathways (Kim et al. 2021). Kim et al. (2021) demonstrated that metabolite profiles of wild-type *S. cerevisiae* at 30°C differ significantly from those of a thermotolerant strain at 42°C. Comparative metabolomics revealed that heat stress markedly affects amino acid and fatty acid metabolism, cell wall integrity (CWI), and membrane composition, all critical to yeast thermotolerance (Kim et al. 2021).

**Figure 1.**
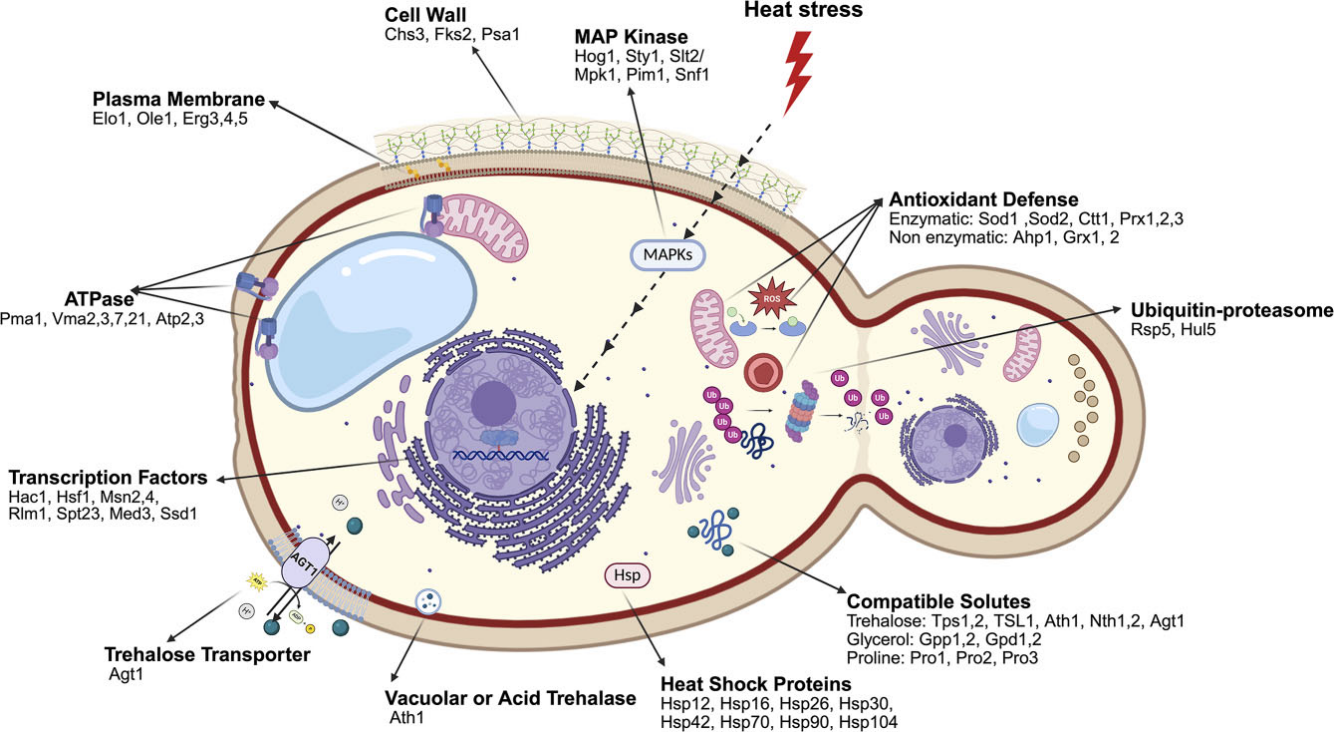
Thermotolerance mechanisms in yeast cells: schematic representation of the components involved in thermotolerance mechanisms.

Here, we classified thermotolerance mechanisms in yeasts into eight primary groups (Table 1 and Fig. 1), including gene expression response, signaling pathways, protection of biomolecules, pH homeostasis, the heat-induced antioxidant defense system, plasma membrane integrity, the CWI, and the ubiquitin–proteasome pathway.

**Table 1.** Summary of cellular mechanisms of thermotolerance in yeast, based on the proteins involved in thermotolerance mechanisms.

Mechanism	Type	Protein	Function	Microorganism (References)
Gene expression response	Transcription factor	Hac1	Binds to UPR-responsive elements in the promoter of UPR target genes	*S. cerevisiae* (Simola 2010, Jarolim et al. 2013, Hou et al. 2014)
		Hsf1	Binds to heat shock elements (HSE) and activate the genes involved in the HSR	*S. pombe* (Saltsman et al. 1999), *S. cerevisiae* (Lagorce et al. 2003, Hahn et al. 2004, Seppä et al. 2004, Bulman and Nelson 2005, Imazu and Sakurai 2005, Yamamoto et al. 2005, Eastmond and Nelson 2006, Hashikawa et al. 2006, Conlin and Nelson 2007, Truman et al. 2007, Chen et al. 2009, Hou et al. 2014, Shui et al. 2015, Caspeta et al. 2016), *C. albicans* (Nicholls et al. 2009, 2011, Brown et al. 2010, Leach and Cowen 2014), *K. phaffii* (Zepeda et al. 2014, Zhang et al. 2018),
		Msn2 and Msn4	The zinc finger proteins can activate STRE-responsive genes	*S. cerevisiae* (Martínez-Pastor et al. 1996, Gasch et al. 2000, Causton et al. 2001, Lagorce et al. 2003, Hahn et al. 2004, Seppä et al. 2004, Yamamoto et al. 2005, Chen et al. 2009, Hou et al. 2014, Shui et al. 2015, Caspeta et al. 2016, Satomura et al. 2016)
		Rlm1	Regulates the expression of genes responsible for maintaining and strengthening the cell wall	*S. cerevisiae* (Dodou and Treisman 1997, Watanabe et al. 1997, Hahn and Thiele 2002, Lagorce et al. 2003, Truman et al. 2007, Eardley and Timson 2020)
		Spt23	Regulates the expression level of Δ-9 desaturase-encoding genes (*FAD9A* and *FAD9B*)	*C. albicans* (Leach and Cowen 2014), *K. phaffii* (Zhang et al. 2018)
		Med3	Related to stress-induced genes and required for thermotolerance	*S. cerevisiae* (Simola 2010)
		Ssd1	Suppressor of *SIT4* deletion, mRNA-binding translational repressor, required for Hsp104-mediated protein disaggregation	*S. cerevisiae* (Mir et al. 2009)
Signaling pathways	MAP kinase	Hog1	The terminal kinase of the HOG pathway is connected to some Msn2/4-regulated genes	*S. cerevisiae* (Rep et al. 2000, Winkler et al. 2002, Seppä et al. 2004), *O. polymorpha* (Kim et al. 2018)
		Sty1	Hog1 homologue in *S. pombe*	*S. pombe* (Chen et al. 2003)
		Slt2/Mpk1	Terminal kinase of the CWI pathway activates genes in response to cell wall stress. It is activated by mild heat shock in response to cell wall weakness detected by the plasma membrane stretch	*S. cerevisiae* (Torres et al. 1991, Kamada et al. 1995, Dodou and Treisman 1997, Gray 1997, Ketela et al. 1999, de Nobel et al. 2000, Hahn and Thiele 2002, Mensonides et al. 2005, Truman et al. 2007, Kim et al. 2008, Guo et al. 2009, Carmody et al. 2010, Varol et al. 2018), *O. polymorpha* (Kim et al. 2018)
		Pim1	Slt2 homologue in *K. phaffii*	*K. phaffii* (Cosano et al. 2001),
		Mkc1	Slt2/Mpk1 homologue in *C. albicans*	*C. albicans* (Kim et al. 2018)
		Snf1	AMP-dependent protein kinase, activates certain genes, deactivates enzymes involved in fatty acid production and glycogen storage, promotes autophagy	*S. cerevisiae* (Bonanomi et al. 2017)
Protection of biomolecules	HSP	Hsp12	Membrane-associated HSP that protects liposomal membrane integrity	*S. cerevisiae* (Martínez-Pastor et al. 1996, Causton et al. 2001, Simola 2010, Welker et al. 2010, Shui et al. 2015)
		Hsp16	Essential for nuclear mRNA export during thermotolerance	*S. pombe* (Chen et al. 2003, Hirose et al. 2005), *O. polymorpha* (Ishchuk et al. 2009)
		Hsp26	Interacts with the protein aggregate, making it accessible to Hsp104. Binds unfolded proteins, preventing their aggregation	*S. cerevisiae* (Causton et al. 2001, Haslbeck et al. 2004, 2005, Cashikar et al. 2005, Franzmann et al. 2008, Auesukaree et al. 2012, Shui et al. 2015, Caspeta et al. 2016)
		Hsp30	A plasma membrane HSP, reducing cellular ATP consumption during stress response	*S. cerevisiae* (Meena et al. 2011, Caspeta et al. 2016)
		Hsp42	The *S. cerevisiae* orthologue of Hsp16. Binds unfolded proteins to prevent aggregation and maintain the solubility of various yeast proteins during heat shock	*S. cerevisiae* (Glover and Lindquist 1998, Causton et al. 2001, Haslbeck et al. 2004, Cashikar et al. 2005, Okuda et al. 2015, Shui et al. 2015, Caspeta et al. 2016)
		Hsp70	Responsible for protein folding, protein degradation, protein translocation to the mitochondria and ER, and protein–protein interactions	*S. cerevisiae* (Glover and Lindquist 1998, Causton et al. 2001, Kimata et al. 2003, Haslbeck et al. 2005, Auesukaree et al. 2012, Lee et al. 2013, Hou et al. 2014, Okuda et al. 2015, Kitichantaropas et al. 2016), *S. pombe* (Chen et al. 2003), *C. albicans* (Nicholls et al. 2009), *K. Phaffii* (Zepeda et al. 2014, Yu et al. 2017)
		Hsp90	Responsible for maturation and regulation of client proteins	*S. cerevisiae* (Truman et al. 2007, Wright et al. 2007, Auesukaree et al. 2012, Kitichantaropas et al. 2016, Fay et al. 2023), *C. albicans* (Nicholls et al. 2009), *K. phaffii* (Yu et al. 2017, Zhang et al. 2018), *S. kudriavzevii* (Fay et al. 2023), *Saccharomyces paradoxus* (Fay et al. 2023), *Saccharomyces uvarum* (Fay et al. 2023)
		Hsp104	Disaggregation of protein aggregates	*S. cerevisiae* (Sanchez and Lindquist 1990, Parsell et al. 1994, Davidson et al. 1996, Elliott et al. 1996, Lindquist and Kim 1996, Glover and Lindquist 1998, Singer and Lindquist 1998, Simola et al. 2000, Causton et al. 2001, Seppä et al. 2004, Cashikar et al. 2005, Haslbeck et al. 2005, Mir et al. 2009, Simola 2010, Lee et al. 2013, Caspeta and Nielsen 2015, Okuda et al. 2015, Fay et al. 2023), *S. pombe* (Chen et al. 2003), *O. polymorpha* (Ishchuk et al. 2009)*, C. albicans* (Nicholls et al. 2009, Leach and Cowen 2014), *K. phaffii* (Yu et al. 2017, Zhang et al. 2018), *S. paradoxus* (Fay et al. 2023)
	Trehalose	Tps1	Trehalose-6-phosphate synthase activates trehalose synthesis in heat stress. It enhances HSP expression, thermotolerance, cell survival, and energy homeostasis under stress	*S. cerevisiae* (Elliott et al. 1996, Singer and Lindquist 1998, Simola et al. 2000, Causton et al. 2001, Mensonides et al. 2005, Conlin and Nelson 2007, da Costa Morato Nery et al. 2008, Mir et al. 2009, Li et al. 2010, Mahmud et al. 2010, Simola 2010, An et al. 2011, Auesukaree et al. 2012, Liu et al. 2014, Saleh et al. 2014, Trevisol et al. 2014), *O. polymorpha* (Amuel et al. 2000), *Wickerhamomyces anomalus* (formerly *Pichia anomala*) (Türkel 2006), *P. farinosa* (Türkel 2006), *Cryptococcus gattii* (Ngamskulrungroj et al. 2009)*, C. neoformans* (Ngamskulrungroj et al. 2009)
		Tps2	Trehalose-6-phosphate phosphatase, activates trehalose synthesis in heat stress	*S. cerevisiae* (Elliott et al. 1996, Causton et al. 2001, Auesukaree et al. 2009, Li et al. 2009, Mahmud et al. 2010, Jarolim et al. 2013, Trevisol et al. 2014, Caspeta et al. 2016), *C. albicans* (Martínez-Esparza et al. 2009), *C. gattii* (Ngamskulrungroj et al. 2009)
		Tsl1	Essential for TPS complex activity	*S. cerevisiae* (Causton et al. 2001, Trevisol et al. 2014, Shui et al. 2015)
		Ath1	Vacuolar or acid trehalase, degrades extracellular trehalose	*S. cerevisiae* (Li et al. 2009, Mahmud et al. 2010), *O. polymorpha* (Ishchuk et al. 2009)
		Nth1	Cytosolic or neutral trehalase, degrades imtracellular trehalose	*S. cerevisiae* (Simola et al. 2000, Conlin and Nelson 2007, Mahmud et al. 2010, Auesukaree et al. 2012, Saleh et al. 2014, Shui et al. 2015), *C. gattii* (Ngamskulrungroj et al. 2009)
		Nth2	Cytosolic or neutral trehalase, degrades imtracellular trehalose	*S. cerevisiae* (Mahmud et al. 2010)
		Agt1	The high-affinity H+-trehalose symporter transports extracellular trehalose into the cell, under normal condition. Under stress, it can reversibly export intracellular trehalose	*S. cerevisiae* (da Costa Morato Nery et al. 2008, Gibney et al. 2015, Shui et al. 2015)
	Glycerol	Gpp1,Gpp2	Glycerol-3-phosphate phosphatase, glycerol biosynthesis	*S. cerevisiae* (Siderius et al. 2000)
		Gpd1,Gpd2	Glyceraldehyde-3-phosphate dehydrogenase have a key role in synthesis of glycerol	*S. cerevisiae* (Siderius et al. 2000, Li et al. 2009), *K. marxianus* (Fu et al. 2019)
	Proline	Pro1	γ-glutamyl kinase, enables proline synthesis	*S. cerevisiae* (Takagi 2008)
		Pro2	γ-glutamyl phosphate reductase, enables proline synthesis	*S. cerevisiae* (Takagi 2008)
		Pro3	Δ1-pyrroline-5-carboxylate reductase, enables proline synthesis	*S. cerevisiae* (Takagi 2008)
pH homeostasis	ATPase	Pma1	Pma1 H^+^-ATPase (P-ATPase), maintaining the structural integrity of cells	*S. cerevisiae* (Coote et al. 1994, Meena et al. 2011)
		Vma2, Vma3, Vma21	Vacuolar H^+^-ATPases (V-ATPase), maintaining pH homeostasis	*S. cerevisiae* (Jarolim et al. 2013)
		Vma7	Vacuolar H^+^-ATPases (V-ATPase), maintaining pH homeostasis	*S. cerevisiae* (Auesukaree et al. 2009)
		Atp2	Beta subunit of the mitochondrial F1F0 ATP synthase	*S. cerevisiae* (Caspeta et al. 2014)
		Atp3	The γ subunit of the mitochondrial F1F0-ATP synthase	*S. cerevisiae* (Caspeta et al. 2014)
The heat-induced antioxidant defense system	Enzymatic defenses	Sod1	Cu/Zn-superoxide dismutase, cytoplasmic, converting the superoxide anion to H_2_O_2_	*S. cerevisiae* (Davidson et al. 1996, Auesukaree et al. 2009, Jarolim et al. 2013, Hou et al. 2014, Xu et al. 2018), *K. phaffii* (Li and Yu 2007)
		Sod2	Mitochondrial Sod2 (MnSOD), protects the mitochondrial electron transport chain and maintains the intracellular redox environment	*S. cerevisiae* (Sugiyama et al. 2000, Hou et al. 2014, Shui et al. 2015, Xu et al. 2018), *O. polymorpha* (Seike et al. 2021)
		Ctt1	Cytosolic catalase T, protects from oxidative damage by H_2_O_2_	*S. cerevisiae* (Davidson et al. 1996, Martínez-Pastor et al. 1996, Hou et al. 2014, Shui et al. 2015, Mejía-Barajas et al. 2017)
		Prx1	Acting as sensors for H_2_O_2_ signaling, protects against H_2_O_2_, can function as both a peroxidase and a molecular chaperone	*S. cerevisiae* (Jang et al. 2004, Shui et al. 2015, Caspeta et al. 2016, Hong et al. 2017)
		Prx2	Protects against H_2_O_2_, helps handle oxidative and nitrosative stresses, can function as both a Trx-dependent peroxidase and a molecular chaperone	*S. cerevisiae* (Jang et al. 2004, Hong et al. 2017)
		Prx3	Eliminates lipid peroxides	*S. cerevisiae* (Hong et al. 2017)
	Nonenzymatic defenses	Ahp1	Alkyl hydroperoxide reductase, reduces hydroperoxides	*S. cerevisiae* (Shui et al. 2015)
		Grx1, Grx2	GSH-dependent oxidoreductases, small heat-stable oxidoreductases	*S. cerevisiae* (Shui et al. 2015)
Plasma membrane integrity	The combination of plasma membrane	Elo1	Medium-chain fatty acyl elongase, enables production of longer fatty acids in heat and chaotrope stress	*S. cerevisiae* (Eardley and Timson 2020)
		Ole1	Δ9 fatty acid desaturase, enables the production of longer fatty acids in heat and chaotropic stress	*C. albicans* (Leach and Cowen 2014), *S. cerevisiae* (Mejía-Barajas et al. 2017), *K. marxianus* (Mejía-Barajas et al. 2017)
	The combination of sterol	Erg3	C-5 sterol desaturase, convert fecosterol to ergosterol	*S. cerevisiae* (Caspeta and Nielsen 2015, Liu et al. 2017, Yang et al. 2023), *K. phaffii* (Zhang et al. 2018)
		Erg4	C-24(28) sterol reductase, catalyses the final step in ergosterol biosynthesis	*S. cerevisiae* (Yang et al. 2023)
		Erg5	C-22 sterol desaturase, convert fecosterol to ergosterol	*S. cerevisiae* (Liu et al. 2017, Yang et al. 2023)
CWI	The structure of the cell wall	Chs3	Chitin synthase III, responsible for the majority of chitin synthesis during stress response	*S. cerevisiae* (Valdivia and Schekman 2003, Pillet et al. 2014)
		Fks2	ß1,3-glucan synthases, involved in cell wall biosynthesis, its expression is induced in response to different stresses	*S. cerevisiae* (Zhao et al. 1998, Kim et al. 2008)
		Psa1	GPD-mannose pyrophosphorylase, involved in cell wall biosynthesis	*O. polymorpha* (Seike et al. 2021)
The ubiquitin–proteasome pathway	Ubiquitin–proteasome	Rsp5	E3 ubiquitin–protein ligase Rsp5, responsible for the increased ubiquitylation induced by heat stress	*S. cerevisiae* (Shahsavarani et al. 2012, Fang et al. 2014)
		Hul5	E3 ubiquitin–protein ligase Hul5, mediating ubiquitination of cytosolic misfolded proteins, required to maintain cell fitness following heat-shock	*S. cerevisiae* (Fang et al. 2011)

### The heat shock sensing mechanisms

The heat shock-sensing mechanisms in yeast comprise a complex set of pathways. In addition to the general stress response, the HSR activates when yeast cells are exposed to elevated temperatures. The HSR is a highly conserved gene expression mechanism that upregulates stress response genes while inhibiting protein production (Gasch et al. 2000, Causton et al. 2001, Eastmond and Nelson 2006, Verghese et al. 2012, Hou et al. 2014, Shui et al. 2015). HSPs are the primary stress response elements (STREs) that are critical for proper protein folding and protection against denaturation. HSP-related genes are regulated by heat shock transcription factor 1 (Hsf1), and their activation is regarded as the focal point of the heat shock-sensing system (Hahn et al. 2004, Wu et al. 2008).

The regulation of Hsf1 activity is a complex process that involves posttranslational modifications, such as phosphorylation and dephosphorylation (Hashikawa et al. 2006, Cho et al. 2014). In response to heat shock, hyperphosphorylated Hsf1 binds to the conserved heat shock element in the promoters of target genes and regulates their transcription (Chen and Parker 2002, Cho et al. 2014). Feder et al. (2021) showed that the J protein (a cochaperone for Hsp70) is depleted from the nucleus under heat stress, leading to Hsf1 activation. It is also shown that the inhibition of proteasomal degradation and, subsequently, the accumulation of misfolded proteins trigger Hsf1 activation (Lee and Goldberg 1998, Trotter et al. 2002). Nevertheless, the temperature threshold for Hsf1 activation is known to be 39°C–40°C, beyond which its activity does not substantially increase (Hahn et al. 2004). Recent studies revealed that Hsp70 can inhibit Hsf1 activity; however, when the level of misfolded proteins increases, Hsp70 separates from Hsf1 through a competitive process, allowing Hsf1 activation (Masser et al. 2019, Ciccarelli and Andréasson 2024). Various stressors, including oxidative stress and ethanol exposure, can affect Hsf1 activation, suggesting a regulatory process extending beyond heat stress alone (Hahn et al. 2004). Cellular factors such as Sir2 (involved in HSR) and Yap1 (mediating oxidative stress responses) can also activate Hsf1 (Nussbaum et al. 2014).

Immediately following heat stress, there is a significant reduction in histone abundance due to rapid nucleosome remodeling (Zhao et al. 2005). The SWI/SNF chromatin remodeling complex and the Rpd3 L deacetylase complex mediate this dynamic process. They are critical for regulating gene expression and the complex interplay between transcription factors and chromatin modifications in HSR (Shivaswamy and Iyer 2008, Alejandro-Osorio et al. 2009, Ruiz-Roig et al. 2010).

In response to heat stress, the zinc finger transcription factors Msn2 and Msn4 hyperphosphorylate and translocate to the nucleus, where they bind to STREs to regulate the expression of general stress response genes (Martínez-Pastor et al. 1996, Garreau et al. 2000, Morano et al. 2012). Msn2 and Msn4 also activate the trehalose biosynthetic pathway, contributing to the induction of the HSR via Hsf1 (Bulman and Nelson 2005, Conlin and Nelson 2007)

Mitogen-activated protein kinase (MAPK) pathways are critical for regulating proteins and transcription factors involved in yeast thermotolerance (Kamada et al. 1995, Qi and Elion 2005, Rodríguez‐Peña et al. 2010, Saini et al. 2018). During the HSR, the essential MAPK pathways are the high-osmolarity glycerol (HOG) and CWI pathways, and their interaction maintains CWI (Dunayevich et al. 2018, Kim et al. 2018). The HOG pathway maintains cellular homeostasis, while the CWI pathway ensures cell wall structural integrity (Dunayevich et al. 2018). Under heat stress, the CWI pathway controls the opening of glycerol channels, allowing glycerol to exit the cell. In response, the HOG pathway, primarily mediated by the MAP kinase Hog1, stimulates glycerol synthesis to restore turgor and maintain osmotic balance, thereby supporting cellular adaptation to thermal stress (Alonso-Monge et al. 2009, Dunayevich et al. 2018).

The mechanism of heat-induced CWI activation remains unclear, but membrane-associated stress sensors such as Wsc1 and Mid2 are involved (Gray 1997, Verna et al. 1997, Rajavel et al. 1999, Kock et al. 2016, Sanz et al. 2017). During heat stress, Wsc1 sensors form clusters (sensosomes) at the plasma membrane, enhancing CWI signaling (Heinisch et al. 2010). Heat stress also upregulates the CWI pathway genes *SLT2* and *FKS2*, which are regulated by both CWI and calcineurin pathways (Zhao et al. 1998, Varol et al. 2018). The CWI pathway, activated by membrane stretch and cell wall stress, relies on its key MAPK, Slt2/Mpk1, which is activated during heat stress (Mensonides et al. 2005, Kim et al. 2008). Under mild heat stress, the Slt2 pathway activates and adjusts membrane fluidity by regulating phospholipid composition (Hahn and Thiele 2002, Leveille et al. 2021). In *O. polymorpha*, for instance, the *MPK1* gene is essential for growth at elevated temperatures, demonstrating the significance of MAPK pathways in thermotolerance (Kim et al. 2018). CWI signaling is also closely linked to the unfolded protein response, a cellular mechanism activated by endoplasmic reticulum stress resulting from the accumulation of misfolded proteins (Kimata et al. 2003, Scrimale et al. 2009, Gardner and Walter 2011).

### Protection of biomolecules

Nearly all species depend on HSPs to survive and recover from heat stress. These proteins primarily act as chaperones, stabilizing proteins, repairing damage, and preventing aggregation during heat stress. Following heat stress, HSPs expression increases dramatically up to 500-fold (Fernandes et al. 2001). Among them, Hsp104 is critical for yeast survival at high temperatures. It operates within an oligomeric complex alongside Hsp40 and Hsp70 to refold denatured proteins and disassemble heat-inactivated proteins from insoluble aggregates (Sanchez and Lindquist 1990, Parsell et al. 1994, Glover and Lindquist 1998, Saleh et al. 2014, Okuda et al. 2015, Yu et al. 2017). Hsp104 also cooperates with trehalose to stabilize the yeast proteome during heat stress (Elliott et al. 1996, Singer and Lindquist 1998, Simola et al. 2000, Saleh et al. 2014). Some HSPs, such as Hsp70 and Hsp90, depend on cochaperones like J-proteins and nucleotide exchange factors to regulate activity and adenosine triphosphate (ATP) hydrolysis (Morano et al. 2012). Small HSPs (sHSPs) bind unfolded proteins to prevent aggregation (Stromer et al. 2003), though their substrates require Hsp70 and Hsp104 for refolding (Haslbeck et al. 2005). In yeast, sHSPs such as HSP26 and HSP42 are essential for maintaining proteome stability during heat shock (Haslbeck et al. 2004).

In yeasts, compatible solutes like trehalose, glycerol, and proline shield biological macromolecules from heat and other denaturation-inducing stresses (Siderius et al. 2000, Zancan and Sola-Penna 2005, Takagi 2008, Shui et al. 2015, Tapia et al. 2015, Dunayevich et al. 2018, Eardley and Timson 2020). Heat stress upregulates trehalose metabolism genes, such as *TPS1* (trehalose-6-phosphate synthase), *TPS2* (trehalose-6-phosphate phosphatase), and downregulates *NTH1* (neutral trehalase) to boost intracellular trehalose concentrations. Trehalose binds to folded proteins to maintain their stability and also prevents the aggregation of nonnative proteins until molecular chaperones can facilitate refolding. It also stabilizes cellular proteins and the cytoplasmic membrane (Singer and Lindquist 1998, Benaroudj et al. 2001, Causton et al. 2001, Saleh et al. 2014, Tapia et al. 2015, Kitichantaropas et al. 2016). Trehalose and glycogen accumulation vary across yeast species. In *S. cerevisiae*, trehalose synthesis is tightly linked to stress adaptation, and mutants lacking trehalose biosynthetic enzymes, such as Tps1, exhibit hypersensitivity to heat, desiccation, and oxidative stress (Silljé et al. 1999). In contrast, genome-wide analysis of *C. albicans* revealed that genes involved in trehalose and glycogen biosynthesis are not strongly upregulated under heat stress (Enjalbert et al. 2003). *ΔTPS1* mutants of *C. albicans* showed reduced resistance to oxidative stress in the presence of glucose, indicating a divergent role for trehalose in this species (Larcombe et al. 2023). In *O. polymorpha*, deletion of the *ATH1* (acid trehalase) gene significantly enhanced heat tolerance. Strains lacking Ath1 exhibited a 12-fold improvement in survival after heat-shock treatment compared to wild-type strains (Ishchuk et al. 2009). However, it was shown that *O. polymorpha* accumulates trehalose only during nitrogen starvation (Türkel 2006).

### pH homeostasis

The V-ATPase and the plasma membrane ATPase (Pma1) are essential proton pumps that maintain a cytosolic pH of ~7.2, particularly under stress conditions. During heat shock, increased membrane permeability permits excess proton influx, resulting in cytosolic acidification (Jin et al. 2022). Pma1 actively expels protons into the extracellular environment to counteract this, while V-ATPases transport protons into vacuoles. The activity of these pumps is regulated by glucose-dependent phosphorylation (Eskes et al. 2018, Jin et al. 2022). Mutations in genes such as *RAS2, IRA1*, and *BCY1* within the Ras–cAMP–PKA pathway reduce ROS accumulation and enhance growth at low pH. These adaptations improve thermotolerance by alleviating glucose repression and optimizing stress signaling (Ribeiro et al. 2022, Salas-Navarrete et al. 2023).

### The heat-induced antioxidant defense system

Cellular stress responses involve multiple pathways, including mitochondrial function. Aerobic respiration may increase during heat shock, elevating ROS production in mitochondria (Sugiyama et al. 2000, Davidson and Schiestl 2001, Fu et al. 2019). This triggers the antioxidant defense system to neutralize rising ROS levels. Enzymatic defenses, including superoxide dismutase (SOD), catalase, and peroxiredoxin (PRX), protect cells from oxidative damage (Davidson et al. 1996, Martínez-Pastor et al. 1996, Sugiyama et al. 2000, Li and Yu 2007, Shui et al. 2015, Gao et al. 2016). Nonenzymatic glutaredoxins (Grx1 and Grx2) also contribute to the heat stress response. Specifically, Grx1 expression increased during both thermotolerance and HSRs, whereas Grx2 was upregulated only during heat shock (Shui et al. 2015). Sakaki et al. (2003) demonstrated that in *S. cerevisiae*, mild heat shock downregulated mitochondrial biogenesis and respiratory function, potentially aiding adaptation to oxidative stress during HSR. Additionally, Nakamura et al. (2014) suggested that genes like *FMP21* are implicated in thermotolerance and protect mitochondria from high-temperature damage.

### Plasma membrane integrity

Yeast cells adapt to fluctuating environments and heat stress through membrane phase separation by protecting proteins involved in key signaling pathways (Leveille et al. 2021, Kimura et al. 2023, Reinhard et al. 2023). Under heat stress conditions, increased levels of ergosterol (the main sterol found in yeast cell membranes) promote the formation of membrane domains, thereby altering the organization and function of the membrane (Abe and Hiraki 2009, Solanko et al. 2018). Additionally, as temperatures rise, yeast cells reshape their vacuole membranes to maintain fluidity and functionality (Leveille et al. 2021, Kimura et al. 2023).

### The CWI

The yeast complex cell wall, composed of polysaccharides, proteins, and lipids, serves as the primary defense against environmental stress. Under heat stress, structural changes in the cell wall modulate intracellular signaling pathways (Chen and Thorner 2007, Levin 2011). In particular, heat stress increases β-1,6-glucan and chitin content while reducing β-1,3-glucan levels to counteract cell wall weakening (Kapteyn et al. 1999, Pillet et al. 2014, Schiavone et al. 2014). In contrast, naturally, thermotolerant yeasts, such as *K. marxianus* and *O. polymorpha*, develop thicker cell walls at elevated temperatures, a trait linked to enhanced structural integrity (Lehnen et al. 2019, Castillo-Plata et al. 2021). For instance, Kim et al. (2018) found that *O. polymorpha* exhibits a thicker β-glucan and chitin layer with longer mannan chains compared to the less heat-resistant species *O. parapolymorpha*.

### The ubiquitin–proteasome pathway

The ubiquitin–proteasome pathway is a critical mechanism for cellular recovery from heat-induced damage by degrading misfolded proteins (Mir et al. 2009, Fang et al. 2011, Shahsavarani et al. 2012). Ubiquitination plays a fundamental role in protein turnover during cell stress, targeting proteins to the 26S proteasome for degradation (Pickart 2000). Ubiquitination influences signaling, cell cycle regulation, and stress responses beyond protein degradation. It also modulates protein function through nondegradative processes (Huang and D’Andrea 2006). This process involves E1 (activating), E2 (conjugating), and E3 (ligase) enzymes, with E3 enzymes facilitating ubiquitin attachment to target proteins. In *S. cerevisiae*, the essential E3 enzymes Rsp5 and Hul5 regulate protein trafficking, degradation, gene expression, and DNA repair under heat stress (Kaida et al. 2003, Krsmanović and Kölling 2004, Fang et al. 2011).

## Conclusion and future perspectives

In light of global warming, thermotolerant yeasts are a promising solution for economically viable and environmentally sustainable bioproduction. These yeasts can improve process efficiency by reducing the need for extensive cooling systems and minimizing contamination risks. This results in lower energy consumption, reduced utility costs, and decreased carbon emissions. Evaluating the heat balance and energy usage involved in using thermotolerant yeasts is needed to reap these benefits. Advances in genetic and molecular biology have improved our understanding of yeast physiology and metabolism, creating new opportunities to harness engineered and evolved thermotolerant yeasts. However, to ensure that their environmental and economic advantages are accurately assessed, more concise sustainability evaluations such as LCA and TEA are also necessary.
